# Women's role in sanitation decision making in rural coastal Odisha, India

**DOI:** 10.1371/journal.pone.0178042

**Published:** 2017-05-24

**Authors:** Parimita Routray, Belen Torondel, Thomas Clasen, Wolf-Peter Schmidt

**Affiliations:** 1 Faculty of Infectious and Tropical Diseases, London School of Hygiene and Tropical Medicine, London, United Kingdom; 2 Department of Environmental Health, Rollins School of Public Health, Emory University, Atlanta, Georgia, United States of America; University of West London, UNITED KINGDOM

## Abstract

**Background:**

While women and girls face special risks from lack of access to sanitation facilities, their ability to participate and influence household-level sanitation is not well understood. This paper examines the association between women's decision-making autonomy and latrine construction in rural areas of Odisha, India.

**Methods:**

We conducted a mixed-method study among rural households in Puri district. This included a cross sectional survey among 475 randomly selected households. These were classified as either having a functional latrine, a non-functional latrine or no latrine at all. We also conducted 17 in-depth interviews and 9 focus group discussions among household members of these three categories of households.

**Results:**

Decisions on the construction of household level sanitation facilities were made exclusively by the male head in 80% of households; in 11% the decision was made by men who consulted or otherwise involved women. In only 9% of households the decision was made by women. Households where women were more involved in general decision making processes were no more likely to build a latrine, compared to households where they were excluded from decisions. Qualitative research revealed that women’s non-involvement in sanitation decision making is attributed to their low socio-economic status and inability to influence the household’s financial decisions. Female heads lacked confidence to take decisions independently, and were dependent on their spouse or other male family members for most decisions. The study revealed the existence of power hierarchies and dynamics within households, which constrained female’s participation in decision-making processes regarding sanitation.

**Conclusions:**

Though governments and implementers emphasize women’s involvement in sanitation programmes, socio-cultural factors and community and household level dynamics often prevent women from participating in sanitation-related decisions. Measures are needed for strengthening sanitation policies and effective implementation of programmes to address gender power relations and familial relationships that influence latrine adoption and use.

## Introduction

Women and girls are the most affected by lack of access to sanitation facilities and safe water[1],as they have greater need for privacy during defecation and bathing compared to men[2]. Absence of sanitation makes females vulnerable and exposes them to the risk of faecal-orally transmitted diseases, uro-genital tract infections, urinary incontinence and chronic constipation[3, 4]. Females avoid being seen while defecating in the day light and wait till dark to use the open space for defecation, which may force them to eat less, resulting in malnutrition[5]. Inadequate sanitation access leads to psychosocial stress, harassment and sexual violence, and increased work from water fetching, care-giving burdens and carrying out post defecation needs of old and ailing family members[6–8]. Provision of adequate water, sanitation and hygiene facilities is thought to mitigate these adverse impacts, making their lives safer, easier and healthier[9, 10]. However, as of 2012, an estimated 1.25 billion women and girls (or 1 in 3 worldwide) were without access to adequate sanitation. Of these, 526 million had no access to any form of sanitation and defecated in the open[10].

In most low-income settings, women and girls are considered to be primary users, providers, and managers of water and sanitation in a household[11]. They are often regarded as guardians of household hygiene, and their inclusion in programmes is believed to be an efficient and sustainable approach to sanitation[1, 6]. Studies have found that the effectiveness of the water and sanitation projects was strongly associated with women’s participation in decisions about water supplies, transparency and management of sanitation interventions[6, 12, 13]. A study in Kenya suggests that if women had the decision making power on major household purchases, then they would influence sanitation improvement[14].Many development programmes acknowledge the need for women participation for their success, and women participation in water and sanitation sector is highly emphasised for the programme’s sustenance[15].

Policies have increasingly emphasized ‘women’ inclusion in sanitation programmes. A few countries in Africa prescribe a minimum percentage of women participation in sanitation interventions and related decision making from the ministerial level to village levels [12]. The Indian government tried addressing the gender inequality in its country wide sanitation programmes—Total Sanitation Campaign (TSC), *Nirmal Bharat Abhiyan* (Clean India Campaign—NBA) and *Swachh Bharat Abhiyan* (Clean India Mission—SBA)[16–18] by reserving 33% membership for women in institutions and bodies related to water and sanitation[16, 18].

However, in actual practice, women’s participation is seldom actively encouraged by the promoters at the field level[19]. Studies have shown that attempts to include women as members in water and sanitation committees, does not guarantee their participation[Routray, 2016 submitted]. Similarly, women attending the community meetings for sanitation promotion and awareness, has not resulted in their participation in community level decision making. Societal and cultural barriers for females, their age, and position within the household are some of the factors, that determine their participation in the sanitation decision making[20–22]. A global review on determinants of latrine ownership in rural households, found a tendency for the final decision to rest with the male head of the household[23]. A study in Ghana found male heads were the decision maker in one in four adopter households, although the whole house owned the toilet [24]. Studies from India show male heads deciding for latrine acquisition, whereas, women were responsible for latrine’s maintenance, keeping the system functioning, fetching water for latrine flushing [6, 22, 25]. There are examples of latrines being acquired by male heads only to secure the privacy and the perceived dignity of the newlywed daughters-in-law, but male heads themselves lacked motivation to use the facility[26–28]. Further, men have been found to be less inconvenienced by the absence of a latrine, and tend to have a lower interest and willingness to install and use sanitary facilities. Thus, low priority among men for sanitation, may result in lower latrine adoption[2].

There are a large number of studies that have analysed female decision making autonomy on different aspects like health, fertility decisions and well-being, and most of them found males taking the decisions[29, 30]. Past research has addressed psychological, economical, social, and environmental determinants of improved sanitation[24, 31]. Studies have also identified behavioural indicators like preference, intention and choice stages for household sanitation decision making[24], with cost stated as a main reason for not constructing latrines[32]. But a recent study on rural Indian population found evidence against this cost proposition. It found that people are defecating in the open not because they are poor, but because they perceived latrines to be expensive[22]. Little research in the field of sanitation is available to inform about women’s autonomy within the households, their participation and contribution, and the household dynamics that could influence women’s ability to contribute to latrine adoption.

This study examines the association between women's decision-making autonomy and latrine adoption in rural areas of Odisha. Decision-making autonomy was assessed in the domains of health care, mobility, small and large purchases, investments and decisions related to household’s latrine acquisition. The paper seeks to answer the following questions—1)Who takes the final decision to build a latrine?; 2) How are the decisions to build a household latrinemade?,3) How do women participate in a household’s latrine installation decisions? 4) Is decision making autonomy associated with latrine adoption?

## Methods

### Ethics (and consent to participate)

The study was approved by the ethics committees of the London School of Hygiene and Tropical Medicine and the local collaborator—the Xavier University. Verbal consent was taken from all the participants of focus groups and interviews. No compensation was paid to study participants. In order to ensure anonymity, names recorded during data collection were deleted, and the analysis was done using household codes. For the questionnaire survey, the participants were explained the study and its objective. Upon consenting to participate, the survey was administered.

### Study area

The study was conducted in rural villages of Puri, a coastal district in the Indian state of Odisha. The villages were also the study villages of a larger randomised controlled trial (RCT) conducted between 2011–2013 and the study setting is described elsewhere [28, 33–35].

### Study design

We conducted a mixed-method study by combining a cross-sectional survey and qualitative research.

### Quantitative study

A survey questionnaire was administered in 12 villages of three blocks—Pipli, Nimapada and Delang in December 2015, that were part of the earlier RCT. From the 12 villages, 6 received the TSC intervention in 2012 and rest received the NBA intervention in 2014. The approaches of these two interventions have been described elsewhere[Routray, 2016submitted]. Pipli was initially chosen as the only block where the survey would take place. On not achieving the targeted sample size from Pipli’s RCT villages, additional villages were included from Nimapada and Delang blocks, and these villages were randomly selected. Within a village, we aimed at recruiting every household in that village, by conducting house to house visits. If a household was not available or declined to participate, we approached the next house. The female head was targeted to be the respondent. Where the female head was either unable or unwilling to participate in the study or not present at the time of the visit, the next household was approached, till all households in the village were covered. Prior to the survey, qualitative research was conducted to understand and identify women’s decision making autonomy in household activities in general. Findings of this qualitative research were used to develop the questionnaire for the cross-sectional survey.

The quantitative cross-sectional survey aimed to capture dimensions of women’s autonomy: 1) decision making power that entailed financial investments such as purchase of large household items, cattle or farm animals, daily needs, repairs or additions to the existing house, and tube-well installation, and, 2) their freedom of mobility in deciding for own health care and accessing health care services, visiting families and friends. The survey also included questions on basic demographics, type and family composition, caste, education and occupation of female and male heads, type of household construction, assets and availability of latrine facility. Decision making in the context of household latrine installation was specifically studied, including aspects such as final decision to build, site identification, purchase of raw materials, arrangement of masons and initial monetary investment. The questionnaire was developed in English, translated to *Odia* (the local language) and then back-translated to assess accuracy. Physical verification of latrine status was done through spot checks; based on which latrines were categorised as functional or non-functional. In order to be deemed functional, the latrine was required to have proper walls, roof, door, a completed pit, and pan not broken/not blocked/and not blocked by leaves.

We aimed to recruit a random sample of atleast 400 households. The sample size was chosen to estimate a proportion of 50% with a margin of error no greater than 5%.The sample size was pragmatically increased to include at least 200 households with a functional latrine and 200 households without any latrine, while maintain random sampling irrespective of latrine ownership or latrine functionality.

### Qualitative study

Members (female and male heads, and other married male and female in the household) from all the three categories of households were selected purposefully based on their availability and willingness to participate in individual in- depth interviews (IDIs) and focus group discussions (FGDs). Seventeen IDIs and 9 FGDs (see Table 1) were held to understand the stages and processes around latrine decision making, and the roles women played in the decision making up till completion of latrine construction. Due to prevailing power hierarchies and social norms that restricted young family members to voice their opinions before the elders, FGDs were held with female and male heads, and male/female family members separated by gender. All the IDIs and FGDs were facilitated by the lead researcher, with the support of a note taker. Additionally, all the IDIs and FGDs were audio recorded and later translated into English for analysis. The lead researcher and the note taker were fully conversant in *Odia*.

**Table 1:** Characteristics of participants in focus groups and individual interviews.

Type	Participants (n)	Gender	Age range (years)	Group type
Focus group 1	8	F	30–45	Female heads
Focus group 2	6	F	30–40	Female heads
Focus group 3	9	F	35–50	Female heads
Focus group 4	8	F	40–55	Female heads
Focus group 5	8	F	25–35	Married younger females
Focus group 6	7	M	40–50	Male heads
Focus group 7	6	M	35–45	Married younger males
Focus group 8	8	M	40–55	Male heads
Focus group 9	8	M	55–65	Male heads
17 Interviews—individuals		M– 8, F– 9	30–65	

Female = F, Male = M

## Data analysis

### Analysis of the quantitative data

For the analysis, households were grouped into 1) owning a functional latrine, 2) owning a non-functional latrine, and 3) not owning any latrine (none of the households shared a latrine with neighbours). These groups were compared pair-wise. Binary and non-ordered categorical variables were compared using the Chi square test. Ordered categorical variables were compared using the Wilcoxon Ranksum Test. Continuous variables were compared using the t-Test. The score test for trend of odds was done to study the association between socio economic status of families and women’s inclusion in latrine installation decision making.

For the analysis of decision making within households, we grouped the various combinations of household members making decisions into three groups: 1) decisions made exclusively by males, 2) decisions involving males and females and 3) decisions made exclusively by females. Data was entered in Epi Info and analysis was performed using STATA version 12.0.

The analysis of the qualitative data was done by thematic ordering and interpretation to identify using Microsoft excel software- 1) the important and active family members taking the final decision to build latrine, 2) processes followed at household level for latrine adoption and 3) factors that favoured or influenced women’s participation in the decision making of the latrine. Each opinion (captured in the form of statements) was highlighted and coded as per the above stated themes. All quotations are in italics and any text within the quote enclosed by square brackets, have been inserted by the authors.

## Results

A total of 475 households were sampled out of which 217had no latrine, 211 had a functional and 47 with a non-functional latrine. The mean number of households was 39.5 per village. Average age of the respondents was 51 years (range = 23 to 86). A total of 2740 individuals lived in the participating households, and the average number of persons per house was 5.8 (range 2 to 16 persons).All the surveyed households practised Hinduism, but belonging to different castes—61% were general caste, 25% were Other Backward Class (OBC) and 14% were Scheduled Castes (SC—lower caste). Seven percent of households were joint families (Table 2).

**Table 2:** Characteristics of respondents (n = 475).

Variables	Variables (categories)	No Latrine (n = 217)	Latrine possession		Total (N = 475) (%)	Functional vs. No latrine (p -value)	Non -Functional vs. No latrine (p -value)
			Non—Functional (n = 47)	Functional (n = 211)			
**Caste**	General,n (%)	137 (63%)	25 (53%)	128 (61%)	290(61%)	0.81*	0.09*
	OBC, n (%)	49 (23%)	9(19%)	59 (28%)	117 (25%)		
	SC, n (%)	31 (14%)	13 (28%)	24 (11%)	68 (14%)		
**Family type**	Joint	16(7%)	2 (4%)	14 (7%)	32(7%)	0.76*	0.44*
	Nuclear	201(93%)	45(96%)	197(93%)	443(93%)		
**Family size**	Mean (SD)	5.4 (2.3)	5.8 (2.6)	6.1 (2.7)	5.8 (2.5)	0.00**	0.32**
**Education of Male Heads**	None (illiterate)	25 (13%)	7 (15%)	13 (7%)	45 (11%)	<0.001***	0.46***
	Primary (1–5 class)	83 (45%)	20 (51%)	59 (34%)	162 (41%)		
	Junior (6–10 class)	68 (37%)	11(28%)	80 (46%)	159 (40%)		
	Senior (11–12 class)	4(2%)	1 (3%)	13 (7%)	18 (4.5%)		
	Graduation/College	5 (3%)	0	7 (4%)	12 (3%)		
	University	0	0	3 (2%)	3(0.75%)		
**Education of Female Heads**	None (illiterate)	99 (46%)	20 (43%)	61 (29%)	180 (38%)	<0.001***	0.87***
	Primary (1–5 class)	72 (33%)	18 (38%)	79 (37%)	169 (36%)		
	Junior (6–10 class)	46 (21%)	9 (19%)	64 (30%)	119 (25%)		
	Senior (11–12 class)	0	0	6 (3%)	6 (1%)		
	Graduation/College	0	0	0	0		
	University	0	0	1 (0.5%)	1		
**Occupation of Male heads**	Farmer	101 (55%)	19 (49%)	99 (58%)	218 (55%)	<0.000*	0.42*
	Share cropper	32 (17%)	10 (26%)	8 (5%)	50 (13%)		
	Labour/Mason	26 (14%)	5 (13%)	13 (8%)	44 (11%)		
	Job (Govt./private)	5 (3%)	3 (8%)	21 (12%)	29 (7%)		
	Business (small)	8 (4%)	1 (3%)	9 (5%)	18 (5%)		
	Business (big)	0	0	1 (0.6%)	1 (0.25%)		
	Unemployed	13 (7%)	1 (3%)	21 (12%)	35 (9%)		
**Occupation of Female head**	Farmer	2 (1%)	0	1 (0.5%)	3 (0.6%)	0.31*	0.55*
	Share cropper	1 (0%)	0	2 (0.9%)	3(0.6%)		
	Labour/Mason	15 (7%)	7 (15%)	5 (2%)	27 (6%)		
	Job (Govt./private)	5 (2%)	0	8 (4%)	13 (3%)		
	Business (small)	4 (2%)	1 (2%)	2 (1%)	7 (1%)		
	Business (big)	0	0	1 (0.47%)	1 (0%)		
	Unemployed	183 (84%)	37 (78%)	183 (87%)	403 (85%)		
	Others	7 (3%)	2 (4%)	9 (4%)	18 (4%)		
**Other Earning members**	Yes	124(57%)	33(70)	151 (72%)	308(65%)	<0.002*	0.10*
	No	92 (43%)	14 (30%)	60 (28%)	166 (35%)		
**HHs monthly income (INR)**	< 5000	106 (49%)	24 (51%)	56 (26%)	186 (39%)	<0.001***	0.87***
	5000–10000	86 (40%)	17 (36%)	108 (51%)	211 (44%)		
	10000–20000	21 (10%)	5 (11%)	26 (12%)	52 (11%)		
	>20000	4 (2%)	1 (2%)	21 (10%)	26 (6%)		
**No. of assets**		2.76 (1.24)	2.78 (0.97)	3.85 (1.47)	3.13 (1.22)	0	0.90**
**Owned house**	Own a house)	215(99%)	46 (98%)	210 (99%)	471 (99%)	0.58*	0.48*
	Did not own	2 (1%)	1 (2%)	1 (0.47%)	4 (0.84%)		
**House built or inherited**	Built self	71 (33%)	12 (26%)	67 (32%)	150 (32%)	0.80***	0.29***
	Inherited	139(66%)	33 (72%)	141 (67%)	313 (67%)		
	Someone else	2 (1%)	1 (2%)	1 (0.48%)	4 (0.86%)		
**House up-gradation**	Major additions	121 (56%)	23 (50%)	142 (68%)	286 (61%)	0.02*	0.42*
	No additions	93 (43%)	23 (50%)	68 (32%)	184 (39%)		
**Own farm animals**	Yes	137(63%)	24(51%)	115 (54.5%)	276(58%)	0.07*	0.12*
	No	80 (37%)	23 (49%)	96 (45.5%)	199 (42%)		
**Own tube-well**	Yes	112(52%)	23(49%)	176 (83%)	311(65%)	<0.000*	0.74*
	No	105 (48%)	24 (51%)	35 (17%)	164 (34%)		
**Own agricultural land**	Yes	147(68%)	30(64%)	179 (85%)	356(75%)	<0.000*	0.60*
	No	70 (32%)	17 (36%)	32 (15%)	119 (25%)		

*-Chi- square test,

**—T—Test,

***—Wilcoxon Ranksum test

The majority of households had male heads. Only in 16% of households, women led after their husband’s death. Very few male heads had higher education in colleges or universities. Percentages of female heads attending senior secondary classes were low compared to male heads (40%). A high percentage (38%) of female heads were illiterate and never went to school. Agriculture was the primary occupation of more than half of male heads. The majority of respondents (85%) were housewives, the rest worked either as agricultural labourers, construction and masonry helpers, had government or a private job, or ran some business.

Compared to households without latrines, households with functional latrines had better educated male and female heads, a larger family size, and higher income. Households belonging to SC tended to have fewer functional latrines than general and OBC families. Family income mostly comprised of the male head’s earnings but in 65% of households (Table 2) other family members such as grown up sons contributed to the income. Households with a functional latrine were more often in higher income categories than households with a non-functional latrine or no latrine, but the difference was only significant in the second group (p<0.001). Households with latrines more often owned agricultural land (85%) and a tube-well (83%) and were less often employed as share-croppers or labourer (Table 2). In contrast, households with non-functional or no latrines had more male heads with an occupation of lower perceived status (working as share cropper, mason or labour) and lower income (p <0.001). Latrine functionality status was associated with the education of the male and female heads (p <0.001). In 56% households that had no latrine and 50% households with a non-functional latrine, major additions to the house were made in the previous two years, suggesting financial capacity for other construction works.

### Decision making of household activities and female’s participation

#### Decision making of household’s different activities

As Table 3shows, the female head along with other females in the family were able to take decisions about their own health care in only 4.4% households. This proportion compares with 11.6% for decisions about visiting family and friends, 3.7% about upgrading the house (or make additions to their existing houses), 10% about tube-well installation, 5.4% about making large household purchases, 20.5% about purchase of farm animals or livestock, and 22.5% about making purchases for daily needs. Females mostly decided what to cook for daily meals, and there was not much involvement of men. The data also shows that women’s non-involvement in the decision making of other important household activities had no strong association with latrine possession or latrine functionality. Even in the 16% households with female heads, males decided for latrine installation in 68% households and the site selection was again done by males in 66% households.

**Table 3:** Women’s involvement in decision making of their own personal lives and household items (n = 475).

Variables	Variables (categories)	No Latrine	Latrine possession		Total N (%)	Functional vs. No latrine (p-value)	Non Functional vs. No latrine (p-value)
			Non- Functional	Functional			
**Determining own health care**	Only males, n (%)	199 (91.7%)	42 (89.3%)	192 (91%)	433 (91.1%)	0.79*	0.56*
	Both groups, n (%)	10 (4.6%)	1 (2.1%)	10 (4.7%)	21 (4.4%)		
	Only females, n (%)	8 (3.7%)	4 (8.5%)	9 (4.3%)	21 (4.4%)		
**Visiting family and relatives**	Only males, n (%)	123 (56.9%)	26 (55.3%)	101 (47.9%)	250 (52.7%)	0.05*	0.82*
	Both groups, n (%)	72 (33.3%)	16 (34.0%)	81 (38.4%)	169 (35.6%)		
	Only females, n (%)	21 (9.7%)	5 (10.6%)	29 (13.7%)	55 (11.6%)		
**Upgrading the house / making additions**	Only males, n (%)	102 (84.3%)	21 (91.3%)	113 (80.1%)	236 (82.8%)	0.37*	0.37*
	Both groups, n (%)	15 (12.4%)	2 (8.7%)	21 (14.9%)	38(13.3%)		
	Only females, n (%)	4 (3.3%)	0	7 (4.9%)	11 (3.7%)		
**Tube-well installation at home**	Only males, n (%)	91 (81.2%)	18 (81.8%)	131 (75.7%)	240 (78.2%)	0.29*	0.97*
	Both groups, n (%)	11 (9.8%)	1 (4.5%)	24 (13.8%)	36 (11.7%)		
	Only females, n (%)	10 (8.9%)	3 (13.6%)	18 (10.4%)	31 (10.1%)		
**Purchase of cattle or farm animals**	Only males, n (%)	25 (65.8%)	5 (83.3%)	24 (70.6%)	54 (69.2%)	0.79*	0.32*
	Both groups, n (%)	5 (13.2%)	1 (16.7%)	2 (5.9%)	8 (10.3%)		
	Only females, n (%)	8 (21.0%)	0	8 (23.5%)	16 (20.5%)		
**Making large household purchases**	Only males, n (%)	65 (82.3%)	19 (90.5%)	91 (75.8%)	175 (79.5%)	0.25*	0.34*
	Both groups, n (%)	11 (13.9%)	2 (9.5%)	20 (16.7%)	33 (15%)		
	Only females, n (%)	3 (3.8%)	0	9 (7.5%)	12 (5.4%)		
**Making purchases for daily needs**	Only males, n (%)	156 (71.9%)	36 (76.6%)	145 (68.7%)	337 (70.9%)	0.46*	0.61*
	Both groups, n (%)	15 (6.9%)	1 (2.1%)	15 (7.1%)	31 (6.5%)		
	Only females, n (%)	46 (21.2%)	10 (21.3%)	51 (24.2%)	107 (22.5%)		

*-Chi square test

#### Decision making of latrine installation and its different components

Table 4 suggests, female’s involvement in decisions regarding sanitation has been minimal. In 9% of households with male heads, females alone had the final say to build the latrine and in 10% households, women participated in the decision for latrine acquisition and installation. For the latrine site selection, in 11% households females exclusively decided, and in 9% households it was a joint decision. In other activities related to latrine installation, such as the purchase of raw materials, arranging masons and investing in latrine construction, female involvement was minimal.

**Table 4:** Women’s involvement in decision making around stages of latrine building(N = 258).

Variables	Variables (categories)	Latrine possession		Total (%) (N = 258)	p -value
		Non Functional (n = 47)	Functional (n = 211)		
**Final say to build a latrine**	Only males, n (%)	41 (87%)	165 (79%)	206 (80%)	0.028*
	Both groups, n (%)	0	27 (13%)	27 (10.5%)	
	Only females, n (%)	6 (13%)	18 (9%)	24 (9%)	
**Latrine site identification**	Only males, n (%)	41 (87%)	165 (78%)	206 (80%)	0.21*
	Both groups, n (%)	1 (2%)	23 (11%)	24 (9%)	
	Only females, n (%)	5 (11%)	23 (11%)	28 (11%)	
**Raw materials purchase for latrines**	Only males, n (%)	10 (83%)	131 (91%)	141 (90%)	0.43*
	Both groups, n (%)	2 (17%)	8 (5.5%)	10 (6%)	
	Only females, n (%)	0	5 (3%)	5 (3%)	
**Arranging masons for latrines**	Only males, n (%)	11 (85%)	134 (92%)	145 (92%)	0.32*
	Both groups, n (%)	1 (8%)	6 (4%)	7 (4%)	
	Only females, n (%)	1 (8%)	5 (3%)	6 (4%)	
**Investing in latrine building**	Only males, n (%)	14 (100%)	119 (91%)	133 (92%)	0.26*
	Both groups, n (%)	0	7 (5%)	7 (5%)	
	Only females, n (%)	0	4 (3%)	4 (3%)	

*-Chi- square test

Table 5 suggests the socio- economic conditions like caste, and education of male and female heads are not associated with female members inclusion in decision making directly. However, in families that had income less than 5000 Indian rupees per month, the female member’s participation in latrine installation decision making was found to be high (30%).

**Table 5:** Association between socio-economic status and women’s decision making to build latrine*.

Variables	N	n	%	p for trend**
**Caste**				
General, n (%)	153	29	19.1%	0.648
OBC, n (%)	68	18	26.5%	
SC, n (%)	37	4	11.1%	
**Male head’s education**				
None (illiterate)	20	5	25.0%	0.127
Primary (1–5 class)	79	5	6.3%	
Junior (6–10 class)	91	22	24.4%	
Senior (11–12 class)	14	2	14.3%	
College/University	10	3	30.0%	
**Female head’s education**				
None (illiterate)	81	17	21.0%	0.509
Primary (1–5 class)	97	15	15.6%	
Junior (6–10 class)	73	17	23.3%	
Senior (11–12 class)	6	1	16.7%	
College/University	1	1	100.0%	
**Family Income (Rupees)**				
< 5000	80	24	30.0%	0.033
5000–10000	125	20	16.1%	
10000–20000	31	3	9.7%	
>20000	22	4	18.2%	
**Female head earning**				
No	219	38	17.4%	0.278
Yes	27	7	25.9%	

*Restricted to households with a latrine. The percentage indicates the share of households where women were involved in the decision making or made the decision alone;

**score test for trend of odds

### Qualitative research: How are latrine decisions made in rural households?

The findings of qualitative data collected through FGDs and IDIs corroborates the quantitative survey results. It shows male heads taking most decisions and women’s participation in all these decisions is minimal. This section describes the stages and the processes involved in the decision, to install latrines in rural houses.

#### Power hierarchies within households

Power hierarchies and the economic status determined the decision making power of the family members: “*After all*, *the husband is the head of the family*, *he is elder in age and in relationship and he will spend for the latrine*, *therefore*, *the decision making power lies with him”*. (IDI—6, Female Head, aged 52). Another participant explains the prominence ‘men’ have in the communities: *“Whatever happens here (in the family or society)*, *it is the father who is looked for*, *and anyone hardly looks for the mother*. *The NGO staff [promoting latrines] also came and asked for men and not us*. (IDI—4, Female Head, aged 62).An earning son and an elderly mother-in-law, had more say than the daughter(s) or daughter- in- law: *“When my sons built the house*, *they informed me*, *but usually males decide*. *Daughter- in- laws are consulted in matters like cooking food*, *purchase of grocery or clothes*. *In matters of expenditures [financial]*, *we usually don’t consult the daughter- in- law”*. (IDI– 8, Female head—aged 70). These indicate about the prevailing power structures (hierarchy) in the communities of Puri.

#### Financial dependency

Money constraint was the most recurring theme through all interviews and focus group discussions and a common reason cited for not opting for latrines, keeping the latrine unfinished and not investing to make the latrine functional. They perceived latrine installation expensive, so men who controlled the household budget, were not keen to build it. Some who had little finances were reluctant to invest in latrines, as they had other priorities.

Many mentioned they depended on the government to build latrines, so waited for subsidies. At the household level, high level of dependency was observed among females on their spouse or guardians (mostly father-in-law) or any earning members in the family (like a son), to decide for activities that had economic implications and this included building latrines: *“If something ‘big’ is to be done for the house that requires more money*, *then my husband*, *who is the family head decides*. *Son(s) join him in the decision*, *as they earn and have more knowledge than me*. *I can only make small purchases like buying a cream or powder [cosmetics]*, *the big ones are to be decided by them*(IDI -5, Female Head—aged 48). Females perceived latrine construction was a ‘big decision’, which only males could take. Even for small purchases, the females relied on their spouse: “*I alone cannot decide*, *we depend on them [husband] for every penny*. *Even for small things like purchasing bangles*, *saree for ourselves*, *we ask them for money*. (FGD 4—women group).

Even when the NGOs approached households to construct latrines (under TSC, where NGOs did the initial spending and constructed the latrines), females would direct the NGO staff to speak to their husband or guardians and explain them the programme and get their approval. Even females with a higher status in the household like the mother—in—law(wife of household head) did not decide themselves, and let their husband and grown up sons (who were earning) to discuss with the NGO and decide. Many female participants mentioned of persuading their husbands for latrines till they get affirmation: “*When the girl [NGO field staff] told us to build a latrine*, *we waited for our husband to come home*. *We would wait for his [husband’s] right mood*, *and initiate the discussion about the latrine*, *otherwise he would get angry*. *They would not instantly agree to our requests*, *as they have to arrange money*, *but we keep on persuading them till they give a nod for it*. *Without their permission*, *we cannot move even a single inch”*. (FGD– 3, Female Head, age range 45–60 years).This suggests that females’ lack of earning, prevented them from making decisions regarding latrines.

#### Gendered roles and perception about female’s abilities

Females had perceptions about their abilities and inabilities to take decisions. Their confinement to the village and the household, made them less confident and doubt their capacities:“*We females don’t know anything*. *All things beyond my house boundaries are done by my husband*, *so they [husband and other males] can decide for the family’s welfare*, *not we”*. (IDI—2, Female Head, aged 42). A male head’s response to the question–‘If they consulted any woman/female in the family, prior to latrine construction?’ corroborate to that of women’s thinking: “*Women are consulted when they either earn or have some education*. *In my home*, *I did not consult anyone*, *when I built the latrine*. *They [women] don't understand many things*, *and have no role to play in latrine construction*. *They [women] needed a latrine*, *which we built” (IDI—10—Male Head aged 55)*. Males also felt superior to women, as is evident from this quote: “*Females roles are cooking*, *taking care of children and doing household chores*. *But*, *when they need money*, *they come to us*, *and we then decide*. (IDI—12, Male head—aged 45; FGD 7 –male group)

In very few households, elderly females were involved in the decisions such as latrine’s site selection: “*My husband decided to build it [latrine] and arranged masons*, *and I was asked to choose the site*. *I then asked other females at home*, *and a commonly agreed site that would be convenient for all was chosen*.(IDI—14, Female Head—aged 65). The younger females also mentioned about their involvement in the site selection: “*When we [daughter- in- laws] placed our demand for a latrine with our husband and father- in- law*, *they agreed to our request but*, *before initiating construction*, *they asked us to locate a suitable place for the latrine*. (FGD 5– Daughter- in- laws; age range 25–40).This indicates, males in a few houses considered women’s views. The survey also found that only a handful of women were involved in the decision to purchase the construction materials for latrine. Most women participants had no prior experience in materials procurement nor had information from where they could get it. They questioned their own capacities: “*Being females*, *can we take all decisions*? *Can we carry the bricks and other materials*? *We even do not know where to get these materials from and how much they would cost”*. (FGD -2, women group).

In a few cases women arranged masons, for example if the mason belonged to the same village and helped procuring the materials. This again indicates that women are relying on men for any kind of purchases. Men made few efforts to engage women: “*For any kind of construction*, *we arrange materials ourselves*, *as we know from where to get them*. *Women have no idea about the market*, *so*, *we did not involve them in such decisions*. *But for digging the pit*, *they sometimes helped* (FGD -8, Men’s group).Overall, in the different components of latrine construction like purchase of raw materials, arranging masons and investments in latrines, females had a very negligible role.

#### Female’s land ownership / entitlement

Families in Puri district were mainly patriarchal. Daughters are not considered permanent members of their natal homes because they become part of their husband’s family after marriage. Women had unequal access to their husband’s parental property, meaning the lands and other properties would be inherited by the husband and not the wife. For example, after the completion of awareness meetings by an NGO, a few motivated females were interested in building a latrine, but could not allow the NGO to do so, as either they had no land or had no direct access to the in—law’s property. They had to seek permission from the father-in-law or husband (whoever is the owner of the land) to build it. Some reported of disputes among siblings regarding parental property division (includes land and other assets), which delayed the decision to build latrine: *“We have plans to build latrine as our daughters are now grown up and are reluctant to go out*, *but we are waiting for the division of the property*. *Once we confirm our share of land*, *we can then decide where to build [the toilet]”* (IDI– 3, Female head, aged—45). Some feared that the latrine might go to the brother-in-law’s [husband’s brother] share after separation, and postponed the construction work. Among families who were landless and had only the homestead land, women who worked as labourers, had some economic power and were able to contribute and participate in household decisions. However, despite having interest in a latrine, many women could not opt for it, due to land unavailability: *“We don’t have any land other than this piece of land where we have our house*. *So*, *even if someone offers to build a latrine for us*, *we cannot do it*. *If someone made land available to us*, *then*, *we might dream to have a latrine*. *We are ready to contribute labour*, *invest some money but*, *the main thing [land] is what we do not have”*. (FGD 5 & 7- women and men groups).

#### Approach by NGOs for toilet promotions

As NGOs were given latrine construction targets to be accomplished by deadlines, their field workers approached mostly the male heads, as getting their permission to construct would be easy, rather than involving women to get permission from their spouse or guardian. In previous paragraphs we described that women lacked the autonomy to decide, and were dependent on their husband or other male members for most decisions. Females complained that the NGOs did not engage them in the process: *“The NGO person looked for the males*. *They had meetings with them [husband and other males]*, *and told us to dig a pit and keep it ready*. *One day*, *they came with a mason*, *and started constructing the latrine*. *He was the only mason to construct all the latrines in the village*, *so*, *due to his unavailability*, *he left the structure unfinished” (*IDI– 14, Female Head, aged 65). Many females expressed unhappiness with their husband’s and NGO’s decision on latrine’s site and mentioned their involvement would have made a much greater difference: “*We cannot use the latrine*, *because it’s placement is wrong*. *It’s built right in front of my house*, *facing the main road*. *How can we use it*, *if it is just on the road*? *This has not only wasted the land but also our house looks ugly with this broken [unfinished] structure at the entrance*. *Had we been engaged in the site selection*, *we would have suggested a better place”*. (IDI 7 –Married Female Head, aged 32). This conveys that latrines failed to give females the privacy they needed. Thus the inappropriate locations of the latrines led people to abandon them: “*All the latrines in our hamlet are built in a line [row]*, *facing our houses*. *It was the sack cement structure and not the concrete walls*. *Half of them are broken now*, *and these latrines are located so close to our house that anyone can see us while using even being inside their house*. *We will not like anyone to see us while defecation*. (FGD 3– Female heads; age range 35–50).

## Discussion

Our results show that prevailing socio-cultural practices, socio-economic constraints, and power hierarchies among household members curtail women’s autonomy regarding their preferences, choices and decision making power with respect to installation of sanitation facility. Women had less education, less exposure to the world beyond their home and village, and little control over resources and finances. This made them less confident, to make sanitation related decisions. Even if females were motivated to install a latrine, they relied on their spouse to take the decision and make arrangements for the construction.

We found latrines were present in households, where the male head had better education and the family’s financial income was higher. But spending on latrine installation or improvement was of least priority to men, often arguing that they had other priorities and financial constraints to build a latrine, which is consistent with the finding of a global review on latrine ownership in rural households[22]. We also found that more than half of households that had a non-functional latrine upgraded their existing houses in the last couple of years, which may indicate, that money was available for upgrading the house but for latrine building. It also indicates rural men not being sensitive to the privacy and security needs of their women[28].

We found women’s involvement in decisions regarding personal as well as household needs was very low, and there is no evidence that their involvement in decision making was greater in households with a functional latrine than with a non-functional latrine or no latrine at all. This signals, despite their varied roles and responsibilities, women often had no voice or choice in the different kinds of services including latrine acquisition. This is a potential constraint on latrine adoption and use in rural India, as is evident from previous research. A survey conducted in multiple states of India found young women who are most likely to use latrines were not the economic decision makers and were least likely to have the intra-household power to allocate resources to building latrine[22]. Similarly, women’s lack of decision-making power in water supply and sanitation projects in India’s Rajasthan state, impacted toilet adoption [25].

Other studies from India report women’s participation in aspects of family decision making like own health care, making daily and major household purchases and socialisation (visiting her family or relatives) to be 37 percent[29], which is much higher than our results. Our findings on women having lower autonomy to decide compared to men on sanitation acquisition, is similar to other studies findings from India on paid work[36], agriculture[37, 38], family planning, pregnancy[39], maternal health care[40] and microenterprises[41]. But, states in India are heterogeneous in nature in terms of geography, environment, community, tradition and culture which are likely to have a significant effect on the nature of female autonomy. For example, women of Meghalaya state, play an important role in the decision-making process in general, as it has a matriarchal society[23]. Similarly, women of Tamil Nadu state have more autonomy in family planning[42].

Female participation and their autonomy in decision making is considered to have positive multiplier effects for the overall social and economic development[43]. In sanitation, their inclusion in the planning and execution has been strongly advocated [6,44, 45]. Some sanitation interventions in recent years attempted motivating both men and women for improved sanitation[31] and focussed on behaviour change in defecation patterns and habits[44, 46]. A drinking water supply and sanitation project in Rajasthan encouraged women to decide on the location of the household latrine[25]. In the TSC implemented villages in Odisha, adolescent girls committee were formed in each village with the purpose to promote usage among household members post latrine construction[34]. A minimum of 33% of seats in the village water and sanitation committees in NBA implemented villages in Odisha were reserved for women [Routray, 2016 Submitted]. A NGO in Odisha state, considered a pioneer in the field of sanitation, trains young unskilled women in masonry—toilet construction and bathing rooms [47]. These examples suggest that measures to improve women participation in sanitation programmes are feasible in rural Indian settings.

However, a majority of the sanitation interventions delivered among the rural Indian communities as observed in TSC, NBA and CLTS, have often not addressed the existing family’s social and political dynamics and gender inequality challenges that determines latrine acquisition[6, 27]. A potential reason could be, NGOs engaged for latrine promotion and demand generation at village level are inexperienced and undertrained[48] [Routray, 2016 Submitted], for which, they fail to address them.

Another aspect of the promotion is the patriarchal messages used for promoting latrine construction, both by national and state media[49]. Most slogans emphasise latrine building so that daughter and daughter-in-laws do not defecate in the open exposing themselves. Such slogans which reinforces the patriarchal ideas and promote only women dignity, might provide sanction to men to continue defecating outside. Therefore such promotions need to be more creative, gender inclusive and not promote patriarchy.

Our study has several limitations. The study was confined to female heads as survey respondents and views of other women family members were not included in the quantitative survey and analysis. In the qualitative study, the subjects were selected purposively, which might incur selection bias. The study did not involve adolescent girls and boys, which could have shed more light on their roles to influence decisions, which however is likely to be minimal. Responses in the quantitative and qualitative parts may be influenced by social desirability or attempts to anticipate what the data collectors supposedly wish to hear. For example, women may have exaggerated their lack of decision making power for latrine construction to hide the fact that their own demand for latrines may be low.

To conclude, the results of this study indicate that males were the explicit decision makers, and only in a few households, females participated or were involved or consulted during the final decision for latrine installation. Lack of control over financial resources was an important factor that limited women involvement in the sanitation decision making. Policies need to be formulated that enable women to participate in the sanitation interventions: We have the following recommendations for sanitation policy makers, planners and the local promoters:

1. Interventions aimed for sanitation promotion and sanitation behaviour change, should be designed to address household level dynamics.
2. Considering the vital roles played by men and women in sustenance of projects, strategies need to be developed to accommodate both gender of different age groups in the decision-making at different stages of sanitation intervention (pre and post latrine construction).
3. The contents of the mass media promotions should not focus on women’s dignity only. Messages should be for both genders and avoid reinforcing patriarchal stereotypes.

## Supporting information

S1 File Discussion guide—Latrine installation and its decision making in rural households of Odisha: “DM—IDI & FGD guidance.docx”. (DOCX)

S2 File Household survey to assess sanitation decision making: “DM—survey.docx”. (XLSX)

S3 File Data set—Sanitation decision making in rural households in Odisha: “DM—dataset—10-02-16.xlsx”. (XLSX)

## Abbreviations

FGD: Focus Group Discussion
GoI: Government of India
HoH: Head of Household
IDI: In-depth Interviews
NBA: Nirmal Bharat Abhiyan
OBC: Other Backward Class
SC: Scheduled Castes
TSC: Total Sanitation Programme
