# Supplementary material for: Women's role in sanitation decision making in rural coastal Odisha, India
Source: PLoS One. 2017 May 24;12(5):e0178042. doi: 10.1371/journal.pone.0178042 (PMC5443550; doi:10.1371/journal.pone.0178042)
Supplement: S1 File — (DOCX) [file pone.0178042.s001.docx]

**DISCUSSION GUIDE**

**RESEARCH TITLE: LATRINE INSTALLATION AND ITS DECISION MAKING IN RURAL HOUSEHOLDS OF ODISHA**

**INDEPTH INTERVIEW – DISCUSSION GUIDE**

**Participant’s name : ....................... .............................. Village : ...........**

**Sex : M/F Age : ........ Marital status : ............. Respondent’s consented : Y/N**

**Date of Interview: ....../......../......... Start time : ................ End time : ....................**

**Introduction /ice breaking questions:**

1. Tell something about your village, your family, friends, and yourself.
2. How is life in rural areas? What are the challenges of living in a village(general) ? What are the good things/what you appreciate about your village?

*Probe : Explore what are the different infrastructure the village has; are any government programmes to improve people’s lives; Any sanitation (latrine) related activities in their village; how united are the villagers; village dynamics; customs and ceremonies*

1. Your own challenges living in this village/in the family?

**Thematic questions:**

1. Has your household participated in any of government’s development schemes?
2. Did your household participate in the recent sanitation programmes of government or in the past?
3. What facilities do you have for defecation/sanitation?
4. Since how long you have this facility at home?
5. Was the facility installed by your household with personal funds or, someone built these facilities for you?
6. If the latrine was built by your household, then who was the initiator of this? What made him to build a latrine/what was his/her motive/idea?
7. Was it a family member or some relative? Any special event, that triggered the family member to build the facility?
8. How were you involved /participated in this latrine installation process?

*Probe: What were your contribution in the whole process of latrine installation, starting from the idea level till completion?*

1. If any external organisation built the latrine for your household, then please describe all the process they followed and built the latrine?

*Probe: Who approached your family? How was the decision taken? How were the sites selected? Who made the investments? Who constructed?*

1. What are the factors that facilitates or constraints this decision in the households?

*Probe: Any household level dynamics; power hierarchies within the family members ; dominance of which members; who has a more strong say in the family and why - economically strong, elderly member, more education ; any special member in the house, who is mostly given importance and why?*

1. If you given an opportunity to build a latrine, how differently you will go about it /do it?

**FOCUS GROUP DISCUSSION GUIDE**

**Total number of Participant: .............................. Village : ........... Group : M/F**

**Age group : ........ Marital status : ............. Consented : Y/N**

**Date of FGD: ....../......../......... Start time : ................ End time : ....................**

**Introduction /ice breaking questions:**

1. Tell something about your village, your family, friends, and yourselves.
2. How is life in rural areas? What are the challenges of living in a village(general) ? What are the good things/what you appreciate about your village?

*Probe : Explore what are the different infrastructure the village has; are any government programmes to improve people’s lives; Any sanitation (latrine) related activities in their village; how united are the villagers; village dynamics; customs and ceremonies*

1. Your own challenges living in this village?

**Thematic questions:**

1. Has your village participated in any of government’s development schemes?

*Probe : sanitation programmes of government or in the past ; rural employment scheme; indira awaw yojana; antodaya; mid day meals, etc?*

1. What facilities the villagers have for defecation/sanitation? Rough estimate (in years) if any villager has the latrine facility.
2. Any external organisation came forward and built the latrine for your households, then please describe all the process they followed and built the latrine?

*Probe: Who approached your family? How was the decision taken? How were the sites selected? Who made the investments? Who constructed?*

1. What are the factors that facilitates or constraints this decision in the households? Share personal experiences.

*Probe: Any household or village level dynamics; power hierarchies within the household and village ; dominance of which type of members (who has a more strong say in the family and why - economically strong, elderly member, more education)*

1. If you had to implement a programme like the sanitation /latrine building, then, how would you approach to household? Or, are you happy the way the programme was delivered?
